# Echocardiographic evaluation of total anomalous pulmonary venous connection: Comparison of obstructed and unobstructed type

**DOI:** 10.1097/MD.0000000000029552

**Published:** 2022-06-24

**Authors:** Yonghua Xiang, Yinghui Peng, Jun Qiu, Qing Gan, Ke Jin

**Affiliations:** a Department of Radiology, Hunan Children's Hospital, University of South China, Changsha, China; b Department of Ultrasound, Hunan Children's Hospital, University of South China, Changsha, China; c House of Journal of pediatric surgery, Hunan Children's Hospital, University of South China, Changsha, China.

**Keywords:** total anomalous pulmonary venous connection, echocardiography, pulmonary venous obstruction, comparison

## Abstract

This study aims to compare the differences between obstructed and unobstructed total anomalous pulmonary venous connection (TAPVC) using echocardiography, and to evaluate the clinical and echocardiographic parameters associated with pulmonary venous obstruction (PVO).

We conducted a retrospective study of 70 patients with TAPVC between 2014 and 2019. The morphologic and hemodynamic echocardiographic parameters of patients were observed and measured, and the parameters between obstructed and unobstructed TAPVC were compared. The clinical and echocardiographic parameter differences between the two groups were used for ROC curve analysis.

Obstructed TAPVC was found in 30 (42.9%) of 70 patients. Between obstructed and unobstructed TAPVC, there were significant differences in atrial septal defect size, pulmonary artery maximum velocity (PA V_max_ ), peak E velocity of mitral valve, left ventricular fractional shortening, left ventricular ejection fraction, stroke volume and the incidence of patent ductus arteriosus, but there was no significant difference in birth weight. The first diagnosis age of obstructed TAPVC was earlier than unobstructed type. The ROC curve analysis for the first diagnosis age showed the sensitivity and specificity were 76.7%, 80% respectively. The ROC curve analysis for the PA V_max_ showed the sensitivity and specificity were 88.5%, 67.6% respectively.

Patients with TAPVC had a high incidence of PVO. The presence of PVO can affect the size of atrial septal defect and the closure of the ductus arteriosus, cause significant changes in PA V_max_, peak E velocity of mitral valve, left ventricular fractional shortening, left ventricular ejection fraction, stroke volume, lead to earlier symptoms and earlier first diagnosis age. The first diagnosis age and PA V_max_ were excellent values since they associated with PVO.

## 1 Introduction

Total anomalous pulmonary venous connection (TAPVC) is an uncommon malformation of congenital heart disease in which all pulmonary veins connect directly to the systemic venous circulation, and not the left atrium (LA). It account for approximately 1% to 3% of congenital heart diseases.^[1,2]^ Pathophysiologically, it was classified into obstructed and unobstructed TAPVC according to whether there was a pulmonary venous obstruction (PVO) in the drainage pathway. Obstructed TAPVC was found in 25% to 50% of patients with TAPVC, and represents a life-threatening neonatal emergency warranting immediate surgery.^[3–5]^ PVO can alter hemodynamics of patients with TAPVC, and then affect the clinical presentation, physiology and prognosis. Thus, the presence of PVO is critical for the timing of surgery and surgical management.^[6,7]^ At present, there are few comparative studies between obstructed and unobstructed TAPVC. The aims of this study were to compare the differences between obstructed and unobstructed TAPVC using echocardiography, and to evaluate the clinical and echocardiographic parameters associated with PVO.

## 2 Patients and methods

### 2.1 Patients

Patients with TAPVC were consecutively collected in Hunan Children's Hospital from 2014 to 2019. A total of 70 patients with TAPVC were included in the study, including 30 obstructed and 40 unobstructed types, 47 males and 23 females, aged from 1 day to 5 years, and median age was 23 days. The discrimination of PVO is determined by CTA. CTA was reviewed by two experienced cardiac radiologist. PVO (obstructed TAPVC) was considered if the area of the draining veins was narrowed by more than 50% in this study. Drainage veins connecting to portal vein were also taken as an indicator of PVO, because obstruction results from the high resistance of portal venous flow needing to traverse through the liver parenchyma before returning to the heart.^[8]^ All patients with TAPVC were diagnosed for the first time after birth. Cases with functionally univentricular circulation, common atrium or pulmonary atresia were excluded. This retrospective study was approved by the institutional review board of Hunan Children's Hospital, and the requirement for informed consent was waived.

### 2.2 Echocardiography

Echocardiographic examinations were performed with PHILIPS EPIQ 7C ultrasound systems equipped with phased array probe (S8-3), including two-dimensional, pulsed wave and color Doppler examinations. All studies were performed by an experienced cardiac echocardiographer and reviewed by another pediatric cardiologist with more than ten years of experience in cardiac imaging. Echocardiographic parameters were measured according to the guidelines of the American Society of Echocardiography (ASE). The morphologic and hemodynamic echocardiographic parameters were recorded as the observation indicators. For parameters affected by age, the ratios were calculated to minimize the effect of age. We calculated the ratio of diameter of pulmonary artery/ascending aorta, right/left ventricular inner diameter and right /left ventricular wall thickness.

### 2.3 Statistical analysis

Data were collected and analyzed with SPSS18. Normally distributed continuous variables were described as mean ± SD. Student *t* tests were used to compare the differences between groups. Descriptive statistics for categorical variables were reported as frequency/percentage and were compared by using of the Chi-Square test (χ^2^). The ROC curve analysis was used to evaluate which variables associated with PVO. Values of *P* < .05 were considered statistically significant.

## 3 Results

**Figure 1:**
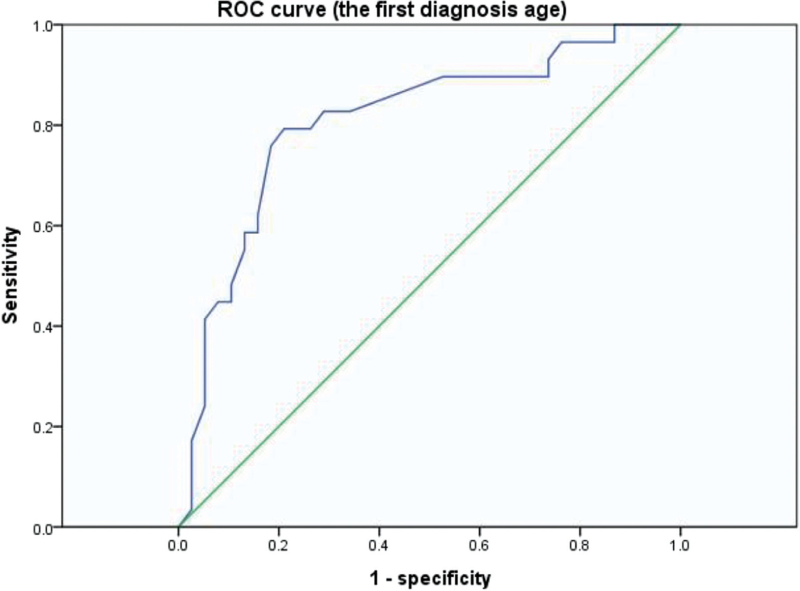
The ROC curve analysis for the first diagnosis age; the area under the curve was 0.805; the sensitivity was 76.7% and the specificity was 80% at the optimal cut-off value of 0.64 months. Smaller parameters represent more definitive test.

**Figure 2:**
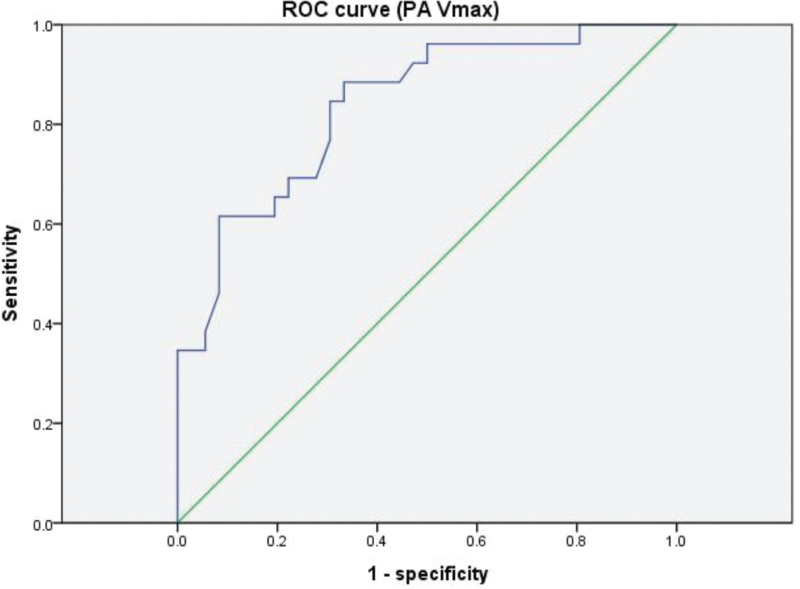
The ROC curve analysis for the PA V_max_; the area under the curve was 0.841; the sensitivity was 88.5% and the specificity was 67.6% at the optimal cut-off value of 1.11m/s. Smaller parameters represent more definitive test.

Obstructed TAPVC was found in 30 (42.9%) of 70 patients in this study. PVO occurred in 22(52.4%) of 42 supracardiac, 0 (0%) of 13 cardiac, 7 (87.5%) of 8 infracardiac, and 1 (14.3%) of 7 mixed types. There was significant difference of the incidence of PVO among the four anatomic types (χ^2^ = 20.149, *P* < 0.001). The first diagnosis age of obstructed TAPVC was earlier than the unobstructed type, and patients with PVO were usually diagnosed during neonatal period (Table 1). The average birth weights of patients with obstructed and unobstructed TAPVC were no different at 3.27 and 3.13 kg, respectively (*t* = 1.573, *P* = .120). Eleven echocardiographic parameters were compared between obstructed and unobstructed TAPVC (Table 2). There were significant differences between obstructed and unobstructed TAPVC in atrial septal defect (ASD) size, pulmonary artery maximum velocity (PA V_max_), peak E velocity of mitral valve (MV V_E_), left ventricular fractional shortening (LVFS), left ventricular ejection fraction (LVEF), stroke volume (SV) and the incidence of patent ductus arteriosus (PDA). These seven echocardiographic parameters with significant difference were used for ROC curve analysis. The results of ROC curve analysis for these parameters were showed in Table 3. The ROC curve analysis for the first diagnosis age (Fig. 1) showed the area under the curve was 0.805, and the sensitivity and specificity were 76.7%, 80% respectively at the optimal cut-off value of 0.64 months. The ROC curve analysis for the PA V_max_ (Fig. 2) showed the area under the curve was 0.841, and the sensitivity and specificity were 88.5%, 67.6% respectively at the optimal cut-off value of 1.11 m/s. The other six echocardiographic parameters appeared to be limited in associating with PVO.

**Table 1:** Comparison of the first diagnosis age between obstructed and unobstructed TAPVC.

		Diagnosis age			
Groups	N	Neonatal (≤28days)	Non neonatal (>28days)	χ^2^ (*P* value)	OR
Obstructed	30	24 (80.0%)	6 (20.0%)	15.52 (.000)	2.46
Unobstructed	40	13 (32.5%)	27 (67.5%)		
Total	70	37	33		

OR = odds ratio.

**Table 2:** Comparison of the echocardiographic parameters between obstructed and unobstructed TAPVC.

		TAPVC (mean ± SD)			
Variables	N	obstructed	unobstructed	T test (or χ^2^)	*P* value
PA/AA	69	1.70 ± 0.24	1.57 ± 0.29	1.979	.052
RD/LD	67	1.55 ± 0.36	1.63 ± 0.36	−0.947	.347
RT/LT	69	1.55 ± 0.45	1.41 ± 0.34	1.402	.167
ASD (mm)	69	7.21 ± 1.91	9.98 ± 4.37	−3.192	.002
PA V_max_ (m/s)	63	0.89 ± 0.32	1.68 ± 1.08	−3.597	.001
AA V_max_ (m/s)	63	0.87 ± 0.19	0.96 ± 0.18	−1.941	.057
MV V_E_ (m/s)	63	0.92 ± 0.16	1.01 ± 0.13	−2.366	.022
LVFS (%)	63	42.3 ± 10.0	36.1 ± 7.1	2.749	.008
LVEF (%)	63	76.1 ± 10.1	68.8 ± 9.2	2.85	.006
SV (ml)	63	2.97 ± 1.63	5.67 ± 3.96	−3.147	.003
Presence of PDA (n, %)	70	21 (70%)	10 (25%)	14.07^∗^	.000

∗ Chi-Square test. AA V_max_ = ascending aorta maximum velocity, ASD = atrial septal defect, LVEF = left ventricular ejection fraction, LVFS = left ventricular fractional shortening, MV V_E_ = Peak E velocity of mitral valve, PA V_max_ = pulmonary artery maximum velocity, PA/AA = the ratio of diameter of pulmonary artery/ascending aorta, PDA = patent ductus arteriosus, RD/LD = the ratio of right /left ventricular inner diameter, RT/LT = the ratio of right/left ventricular wall thickness, SV = stroke volume, TAPVC = total anomalous pulmonary venous connection.

**Table 3:** The ROC curve analysis of the clinical and echocardiographic parameters associated with PVO.

Predictors	Cut-off points	Sensitivity (%)	Specificity (%)	Areas un-der curve	95% CI	*P* value
ASD (mm)	8.85^∗^	82.8	55.0	0.707	0.584–0.830	.003
PDA (exist = 1)	1	70.0	75.0	0.725	0.602–0.848	.001
PA V_max_ (m/s)	1.11^∗^	88.5	67.6	0.841	0.744–0.939	.000
LVFS (%)	40.5^♯^	54.2	78.8	0.658	0.509–0.808	.043
LVEF (%)	75.5^♯^	54.2	78.8	0.668	0.520–0.816	.032
SV(ml)	3.1^∗^	70.8	75.8	0.751	0.622–0.879	.001
MV V_E_ (m/s)	0.925^∗^	51.9	82.9	0.671	0.532–0.831	.021
Diagnosis age (mo)	0.64^∗^	76.7	80.0	0.805	0.698–0.912	.000

CI = confidence interval, ROC = receiver operating characteristic, PVO = pulmonary venous obstruction.

∗ Smaller parameters represent more definitive test.

♯ Larger parameters represent more definitive test; Mon, months. ASD = atrial septal defect, LVEF = left ventricular ejection fraction, LVFS = left ventricular fractional shortening, MV V_E_ = Peak E velocity of mitral valve, PA V_max_ = pulmonary artery maximum velocity, PDA = patent ductus arteriosus, PVO = pulmonary venous obstruction, SV = stroke volume.

## 4 Discussion

### 4.1 Incidence and location of PVO

TAPVC was classified into supracardiac, cardiac, infracardiac and mixed types according to the drainage site of the pulmonary veins. The incidence of PVO was highest in the infracardiac and lowest in the cardiac type.^[9,10]^ Generally, the longer of the drainage vein connecting to right atrium (RA), the higher incidence of PVO. The infracardiac and cardiac types have the longest and shortest drainage vein respectively of four types. In this study, PVO occurred in 87.5% of infracardiac, 52.4% of supracardiac, but none of cardiac type (0%). In supracardiac, the most common sites of PVO were at the level of the vertical vein (VV), especially at the place where the VV step over the left pulmonary artery (PA) and bronchus, and the VV was often oppressed by them. In infracardiac, the drainage veins were usually connected to portal vein (PV), and obstruction resulted from the high resistance of PV flow needing to traverse through the liver parenchyma before returning to RA.^[8,11,12]^

### 4.2 Prenatal and postnatal physical development of patients with TAPVC

Our study showed the patients with TAPVC had normal birth weight, and there was no significant difference in birth weight between obstructed and unobstructed TAPVC. The reason was the unique nature of fetal hemodynamics allows the fetus with TAPVC to be well tolerated in utero. Pulmonary blood flow of the fetus was a small portion of the combined ventricular output, so the physical growth of the fetus will not be affected in utero. However, there was complete mixing of the pulmonary and systemic circulations in the right heart after birth. The patients will be cyanotic and may have difficulty feeding in the first weeks or months of life. If there was a PVO, it resulted in elevated pulmonary pressures and decreased systemic oxygen delivery, and severe respiratory distress and decompensation may ensue very early in the neonatal period. The patient's conditions can deteriorate rapidly and become life threatening unless the cause was promptly recognized and treated.^[13]^ Our study demonstrated that the first diagnosis age of obstructed TAPVC was earlier than the unobstructed type, and patients with PVO were usually hospitalized during neonatal period. We believed that the presence of PVO led to earlier symptoms and thus to be discovered earlier.

### 4.3 Influence of PVO on ASD size and the closure of ductus arteriosus

For patients with TAPVC, a right-to-left intracardiac shunt is obligatory for survival, and it almost always occurs at atrial level through an ASD that is rarely restrictive. ASD size is one of the most important factors affecting the survival period. The presence of a large ASD allows some of the excessive venous return in RA to reach LA. Several cases of adult untreated TAPVC have been reported, even a few cases were diagnosed after 50 years of age, and they have very similar pathophysiology to a large ASD.^[14–16]^ All our patients presented ASD of various sizes except a case with common atrium, and unobstructed TAPVC had a larger ASD size than obstructed type. The first diagnosis age of unobstructed TAPVC was later than obstructed type, so a larger ASD size maybe resulted in later diagnosis age. PDA was the common concurrent abnormality with an incidence of 44.3% in this study. The direction of PDA flow was right to left or bidirectional shunt in 26 of 31 patients. We found that obstructed TAPVC had a higher incidence of PDA than unobstructed type, and the patients with PDA had a smaller ASD than that without PDA. The presence of PDA may relate to pulmonary pressure. Obstruction of drainage vein caused elevated pressure in pulmonary venous territory, elevated pressure in pulmonary capillary bed, pulmonary edema, pulmonary hypertension, and right heart failure with leftward shift of the interventricular septum and low systemic output. A PDA can alleviate the pulmonary hypertension and provide more blood flow for systemic circulation.

### 4.4 Influence of PVO on echocardiographic parameters

Pulmonary arterial blood flow velocity was related to right ventricular function, pulmonary arterial pressure, blood flow and arterial diameter and so on. Right ventricular volume overload and pulmonary hypertension were common findings in patients with TAPVC,^[17]^ and patients with TAPVC were often found having abnormal pulmonary artery flow velocity. Our study demonstrated that PA Vmax significantly higher in patients with unobstructed TAPVC than obstructed type. Obstructed type had a lower PA Vmax, which may be resulted from increased pulmonary vascular resistance and decreased compliance due to obstructed drainage veins. However, unobstructed type had an accelerated PA Vmax, we speculated that the increase of pulmonary circulation blood flow was not compatible with the expanding pulmonary artery itself, resulting in functional pulmonary artery stenosis, then led to an accelerated PA Vmax.

Enlargement of the right heart was a common feature to patients with TAPVC. The right ventricle was disproportionately larger than the left ventricle (LV) in most of our cases. The LA was frequently small and the LV was compressed by the dilated right ventricle. The ratios of the ratio of right /left ventricular inner diameter of obstructed and unobstructed TAPVC were larger than normal, but there was no significant difference between obstructed and unobstructed type. For patients with TAPVC, the blood of left heart was almost always obtained by an ASD, so the size of ASD could often affect the LV volume. The presence of PVO reduced the blood flow returning into the RA, the obligatory right-to-left shunt was also attenuated and the LV volume became small. The obstructed TAPVC had a smaller ASD than unobstructed type, which resulted in decreasing of SV in patients with obstructed TAPVC. Our study demonstrated that the obstructed TAPVC had a higher LVFS and LVEF than unobstructed type. The main reason may be that obstructed TAPVC need to increase cardiac output through strengthening the ventricular systole.

### 4.5 Clinical and echocardiographic parameters associated with PVO

TAPVC has an excellent outcome if detected early and corrected surgically in due time, but the prognosis is poor when TAPVC is associated with a PVO.^[18]^ The presence of PVO was significantly associated with an increased risk of death.^[3,7]^ Therefore, an important part of preoperative diagnosis of TAPVC was to determine whether there was a PVO. Echocardiography can detect the PVO by measuring the blood velocity of the stenotic pulmonary vein.^[19]^ We tried to detect PVO by some familiar, easily obtained echocardiographic and clinical parameters. The first diagnosis age of obstructed TAPVC was earlier than the unobstructed type. The ROC curve analysis for the first diagnosis age showed the area under the curve was 0.805, and the sensitivity and specificity were 76.7%, 80% respectively at the optimal cut-off value of 0.64 (months). Therefore, we can make a preliminary judgment whether there was a PVO according to the first diagnosis age, which was the easiest parameter to obtain. If the first diagnosis age was less than 0.64 months, then a suspicion of PVO was strongly suggested, and further imaging examination was necessary. For both clinicians and echocardiographer, this was a convenient and quick way to understand the patient's conditions. Among echocardiographic parameters, the PA Vmax was also an excellent value in detecting PVO. It had 88.5% sensitivity and 67.6% specificity at the optimal cut-off value of 1.11m/s, and the area under the curve was 0.841. The other parameters appeared to be limited in its detection ability.

### 4.6 Study limitations

Some limitations should be addressed in this study. First, TAPVC is classified into multiple anatomical types, and the PVO positions of different types are different, which may affect the hemodynamics of patients with TAPVC. Due to the sample size of this study is small, there is no further study based on anatomical classification. Second, the degree of PVO has an impact on hemodynamics. Similarly, due to the small sample size, there is no further study based on the degree of PVO. Third, although we have tried our utmost to control the influence of age factors on parameters, it still could not be completely eliminated.

## 5 Conclusions

Patients with TAPVC had a high incidence of PVO, especially in the infracardiac type; whereas, cardiac type was less likely to obstruct. The physical growth of the fetus with obstructed or unobstructed TAPVC will not be affected in utero. After birth, the presence of PVO in patients with TAPVC can affect the size of ASD and the closure of the ductus arteriosus, cause significant changes in PA V_max_, peak E velocity of mitral valve, LVFS, LVEF, SV, lead to earlier symptoms and earlier first diagnosis age. The first diagnosis age and PA V_max_ were excellent values since they associated with PVO.

## Author contributions

Ke Jin: Conceived and designed the experiments. Yonghua Xiang: Analyzed the data and wrote the paper. Yinghui Peng, Qing Gan and Jun Qiu: Collected and analyzed the data. All authors have read and approved the manuscript.
